# A Study on the Competence Characteristics of Psychological Hotline Counselors During the Outbreak of COVID-19

**DOI:** 10.3389/fpsyg.2021.566460

**Published:** 2021-05-07

**Authors:** Linyu You, Xiaoming Jia, Yaping Ding, Qin An, Bo Li

**Affiliations:** School of Humanities and Social Sciences, Beijing Institute of Technology, Beijing, China

**Keywords:** COVID-19, psychological hotline, psychological counselors, competency characteristics, Chinese experience

## Abstract

**Introduction:** After the outbreak of COVID-19, psychological hotlines functioned as a main channel of psychological assistance and required a large number of professionals to provide services. These hotlines mostly offered a single-use service with short session times and allowed callers to retain anonymity. They functioned as a psychological counseling service for stress experienced in the COVID-19 public health emergency. Hotline psychological counselors must meet special competency requirements. The selection and evaluation tools for recruiting hotline counselors need to be developed.

**Materials and Methods:** The initial scale of competence for psychological hotline counselors was formed by expert evaluation based on theoretical constructs and by using the Delphi method. A link to the questionnaire was sent to a WeChat group of counselors from 36 major psychological hotlines in China in two stages. The questionnaire consisted of questions to elicit basic demographic information and the initial competence scale. In the first phase, 343 valid samples were used to perform exploratory factor analysis. In the second phase, 334 valid samples were used to perform confirmatory factor analysis. The status of the competence of psychological hotline counselors was also analyzed.

**Results:** The factor structure of the Psychological Hotline Counselor Competence Scale was verified and defined in terms of skills, attitude, and knowledge. The results of exploratory factor analysis and confirmatory factor analysis showed that the scale has good reliability and validity (χ^2^/df = 1.758, GFI = 0.86, RMSEA = 0.05, CFI = 0.96, NFI = 0.91, NNFI = 0.95). The McDonald’s omega for each factor was calculated (ω_F__1_ = 0.927, 95%CI [0.914, 0.940]; ω_F__2_ = 0.958, 95%CI [0.951, 0.965]; ω_F__3_ = 0.954, 95%CI [0.945, 0.961]). Meanwhile, it was found that the psychological hotline counselors’ self-assessed competence had a high average score (*n* = 334).

**Conclusion:** The Competence Scale for Psychological Hotline Counselors for Major Public Emergencies developed in this study has good reliability and validity, and can be a reliable tool for organizing psychological assistance and screening hotline psychological counselors during public emergencies in the future.

## Introduction

The global outbreakof the novel coronavirus (COVID-19) was a public health emergency. Due to the highly contagious nature of COVID-19, all psychological assistance was provided remotely (via hotlines or online). During the epidemic, many new hotlines were established in various parts of China, including Wuhan (The State Council, 2020). To provide psychological assistance, the Psychological Assistance Platform of the Central China Normal University of the Ministry of Education alone recruited nearly 3,000 psychological counselors as volunteers. As the main contact point for psychological assistance during a major public health emergency, the psychological hotlines should have specific requirements for the competencies and qualifications of the hotline counselors (National Health Commission of the People’s Republic of China, 2020). However, to date there is a lack of criteria for recruiting competent counselors for psychological assistance hotlines.

McClelland (1973) first proposed the concept of competence. Competence means being able to work according to corresponding professional standards (Barnett et al., 2006). In the field of psychological counseling, it requires counselors to possess the knowledge, skills, and abilities needed, and to practice them ethically to provide effective services (Barnett and Johnson, 2008). The competence of psychological counselors involves ethics and the law. Professional associations worldwide stipulate in their code of ethics that psychological counselors and psychotherapists must have professional competence (American Counseling Association [ACA], 2014; American Psychological Society [APA], 2017; Chinese Psychological Society, 2018), and believe that a lack of competence is often the main cause of harm to clients (Corey and Corey, 2011).

However, establishing criteria for evaluating competence is a complex and difficult issue (Kitchener, 2000, pp. 154–155). Spencer and Spencer (1993, p. 324) noted that “obvious traits such as knowledge and skills are the benchmarking characteristics of competence, and implicit traits such as attitudes and values are distinctive competence characteristics.” Kaslow (2004) pointed out that the assessment of professional competence should target all areas of competence and related knowledge, skills, and attitudes (McIlvried and Bent, 2003; Kaslow, 2004).

Assessment of counseling trainees’ competencies focuses on specific skills, and is measured through tools such as the Counseling Skill Scale (Eriksen and McAuliffe, 2003). The APA Benchmarks Work Group (Fouad et al., 2009) developed a Competency Benchmarks document and outlined the core foundational and functional competencies required for professional psychologists at different levels. However, only 48% of the APA-accredited programs adopted it (Grus et al., 2016). Researchers suggested that it would be better to integrate the Competency Benchmarks into routine supervision as they just provided an assessment framework rather than a verified inventory. Recently, Lambie et al. (2018) refined the Counseling Competencies Scale (Swank et al., 2012) and reported relatively sound reliability and validity; this scale was mainly used in the assessment of doctoral students’ practicum and internships.

Some researchers in China have indicated that the core competencies of psychologists and therapists should include six aspects: professional attitudes and behaviors, knowledge on ethics and law; clinical knowledge and skills;, science and research; relationship-building skills; multicultural and Chinese cultural awareness; and case management (Wang M. et al., 2015). Research on the competence of psychological counselors includes the study of competency characteristics, such as those of group counselors (Xiao et al., 2016). The other focus of the research is the development of competency inventories, such as those for mental health personnel (Zhang, 2011) and school psychological counselors (Xie, 2008).

The question emerges as to whether there are specific competence requirements for psychological hotline counselors. Ordinary psychological hotline counselors have always been recruited as volunteers (Jia and An, 2006, pp. 22), and there are no strict requirements regarding professional and academic background. Before they take up their jobs, they receive unified training and belong to the field of quasi-professionals. Research on psychological hotlines has focused on an analysis of the characteristics of callers, including help-seeking problems and personal characteristics (Wang C. L. et al., 2015; Chen and Yang, 2016), analysis of the hotline consultation process (Qin and Jia, 2015; Yu and Li, 2015), the effect of hotline intervention (Wang et al., 2011), and the organization and management of hotlines (Cui et al., 2016). Psychological research on the use of hotlines after public health emergencies also focuses on the characteristics of help-seeking problems (Xu and Jiao, 2003; Zhou and Wang, 2004). These studies do not focus much on the competence of hotline counselors.

In a recent study, Nie et al. (2019) interviewed clinical psychologists with experience in disaster relief psychological assistance to clarify the required competencies of psychological assistance personnel. All interviewees believed that not all counselors are able to provide disaster relief psychological assistance. They generally emphasized the importance of mastering the knowledge and skills related to psychological rescue, hours of consultation, hours of supervision, and personal experience.

In contrast to the previous use of psychological hotlines, the psychological assistance hotlines that opened during the COVID-19 epidemic involved previously offline psychological assistance being moved to the phone line. The professional work of psychological counseling had to change its mode of operation. However, in comparison to offline services, hotline work has unique features such as a short session time, mostly one-time consultations, a certain degree of anonymity, and voice-only communication. Although most volunteers of psychological assistance hotlines are psychological counselors with professional qualifications, they still need to have corresponding professional competence (Jia and An, 2020). In particular, there were specific psychological problems caused by the sudden public health event of the COVID-19 epidemic. People experienced psychological stress in the early stage of the epidemic, grief reaction caused by loss in the middle and late stages of the epidemic, and post-traumatic stress disorder. There were economic pressures, family conflicts, and other problems as a result of the epidemic (Li et al., 2020; Su et al., 2020; Xu et al., 2020). As such, the professional competence of the hotline psychological counselor in the epidemic situation requires specific investigation.

In China, most of the counselors recruited by the psychological assistance hotlines during the COVID-19 epidemic were certified and had some qualifications. They were certified by different institutions, and had received different training and supervision. Their experience varied widely. Currently, there is no instrument to assess the competence of hotline psychological counselors. This also brings some difficulties for selecting counselors in emergencies. There are different competence models for psychological counselors in general situations. The characteristics of the hotline and the nature of emergencies should also be considered in epidemics. Psychological assistance for public health emergencies is indispensable, and competent hotline counselors are the key to the quality of psychological services provided. Therefore, it is necessary to study the characteristics of the competence of hotline counselors and develop a corresponding assessment scale. This scale can be used for screening, training, supervising, and assessing psychological assistance professionals during public health emergencies in the future.

## Materials and Methods

### Theoretical Construction: Preliminary Construct of the Competence of Psychological Hotline Counselors Based on the Competence Model

A psychological counselor’s professional competence is a basic requirement for counseling practice. It generally includes a collection of traits including knowledge, ability, and attitude (Halley, 2001). Existing psychological counselor competence scales differ from one another and lack uniformity (Xie, 2008; Zhang, 2011; Xiao et al., 2016), but the theoretical construction of the scales has not gone beyond skills, attitudes, and knowledge. Knowledge means systematic study of the history, theory, and research in the field (Welfel, 2010). It includes being aware of which knowledge and intervention methods should be applied in specific situations and having objective standards to evaluate theory and research (Spruill et al., 2004). Skills refer to the ability of professionals to judge which intervention method is the most appropriate under current circumstances based on current counseling and treatment research (Welfel, 2010). Attitudes usually include aspects such as ethics and values, which means that the client’s needs are the first priority for the counselor, who tries his or her best to help the client. If a counselor is not able to help the client, then he or she must be willing to refer the clients on to others (Welfel, 2010). Regarding the specific content of competence, the core competencies of counselors and therapists in China include professional knowledge and behavior; knowledge on ethics and law, science, and research; clinical knowledge and skills; relationship-building skills; multicultural and Chinese cultural awareness; and case management (Wang M. et al., 2015).

Sandberg (2000) emphasized that assessment of competence should fully consider the specific function of the work situation. Due to the high level of infectiousness and the wide-ranging impact of the COVID-19 epidemic, all psychological assistance could only be administered in the form of a hotline. Counselors participating in psychological assistance may also face psychological crisis interventions caused by the epidemic. Wu and Sang (2010) developed a competence model that focused on distinctive competency and threshold competency, and proposed a preliminary construct for the competence of psychological hotline counselors accordingly. The construct included the basic competencies of psychological counseling (threshold competency) and the competency requirements based on special circumstances (distinctive competency). The latter included both the competencies needed for general psychological hotlines and the required competencies for giving psychological assistance in major public emergencies.

In this study, the researchers analyzed the job responsibilities and work tasks of psychological hotline counselors in the epidemic situation, and applied the competence model to assess the competency characteristics of the hotline psychological counselors. The competence requirements for counselors operating the psychological hotline and giving emergency psychological assistance in terms of knowledge, skills, and attitudes are emphasized (Table 1). In order to meet the requirements of three aspects, 100 items were prepared for the preliminary scale.

**TABLE 1:** The primary construct of the competence model for psychological hotline counselors.

Domain	Threshold competency	Distinctive competency
Knowledge	Basic knowledge of counseling	Basic knowledge of psychological hotlines
		Basic knowledge of public health emergencies
Skill	Basic skills of counseling	Basic skills of psychological hotline counseling
		Basic skills of crisis intervention
Attitude	Ethics for counseling	Ethics for psychological hotline counseling
		Ethics for crisis hotlines

### Preliminary Competence Scale

Using the Delphi method, the preliminarily developed measurement questions were distributed to eight psychological experts to seek their opinions on each question. The expert members included six females and two males, all registered counseling supervisors of the Chinese Psychological Counselor Registration System with over 20 years supervision experience. They participated in SARS psychological relief, provided psychological assistance after the Wenchuan earthquake on May 12, 2008, and provided psychological assistance in other major domestic emergencies. They, therefore, had a vast amount of practical experience and also served as hotline supervisors during the COVID-19 epidemic.

After obtaining the experts’ preliminary opinions, the measurement scale was modified according to their feedback. The modified measurement questions were sent to the expert group again to obtain further suggestions. After two rounds of feedback, the project team aggregated the experts’ suggestions to form a first draft of a 57-item questionnaire with three dimensions. There are 13, 22, and 22 items, respectively, on the dimensions of knowledge, skills, and attitude. Examples of the items include “understanding the advantages and limitations of hotline consulting,” “able to respond flexibly to emergencies in the hotline,” and “ability for self-reflection after each hotline consultation.” A 5-point Likert scale was used to identify the degree to which the item description was consistent with the participants: 1 = completely inconsistent, 2 = inconsistent, 3 = neutral, 4 = consistent, and 5 = completely consistent.

### Questionnaire Distribution and Data Collection

The questionnaire included the first draft of the competence scale, items on demographic information, and items on professional experience and training. The questionnaire was randomly distributed to psychological counselors at 36 well-established hotlines in China. It was distributed by forwarding the questionnaire link to the WeChat groups of the hotlines during two periods. The first data collection period was from March 15 to March 20, 2020 (Sample A). The preliminary measurement scale was administered to participants. The data from this period were used for exploratory factor analysis (EFA) and item analysis. The second data collection phase was from March 22 to March 28, 2020 (Sample B). The revised scale based on EFA was distributed to collect data for verifying the reliability and validity of the scale.

The questionnaire was distributed through the Internet and quality control was carried out in three ways. First, the integrity of the data was confirmed by deleting data from incomplete questionnaires. The second consideration was response time: samples with short response times were excluded. Third, data with unclear basic information and from questionable sources was screened out.

### Data Analysis

The data were analyzed using SPSS 19.0 for exploratory factor analysis (EFA) and AMOS 20.0 for confirmatory factor analysis (CFA). First, we conducted EFA with Sample A (*n* = 343). The KMO test (>0.8) and Bartlett’s test of sphericity (*P* < 0.001) were conducted to examine whether the variables were suitable for factor analysis. The criteria for item elimination were as follows: commonality (common factor variance) less than 0.4, or a serious deviation from the corresponding relationship of the factors (the load coefficients on two or more factors are relatively close to each other). The second EFA was conducted with the following criteria for item retention: (a) the eigenvalues of the three factors were all greater than 1; (b) a factor loading of 0.5 or greater. Next, according to the frequency distribution of the total score of each sample, all samples were divided into a high-score group (P71, since eight samples had equal score ranking from P71 to P73) and a low-score group (P27). Item analysis using the independent sample *t*-test was conducted. Third, point and interval estimates of McDonald’s omega were calculated in R (R Development Core Team, 2012), using congeneric models along with bootstrapping to obtain confidence intervals for omega (Raykov, 1998). Guttman’s half-reliability coefficient was also calculated to examine the reliability. Finally, CFA was conducted to examine the factor structure and the χ^2^ ratio (χ^2^/df < 3), GFI (>0.9), RMSEA (<0.1), RMR (<0.05), CFI (>0.9), NFI (>0.9), and NNFI (>0.9) were calculated.

## Results

### Exploratory Factor Analysis

Among the 343 participants of Sample A, 85.4% were female (M_age_ = 43.2 years, S_age_ = 9.5). Subjects with an education level of master’s degree, undergraduate degree, and college diploma and below comprised 41.7, 49.6, and 8.7% of the sample, respectively.

The statistical results showed that the sampling suitability index KMO value was 0.965; therefore, it was appropriate to use factor analysis to verify the validity of the scale. According to the results of the EFA, 17 items were deleted and a 38-item hotline counselor competence scale was developed. After removing the data associated with 17 deleted items, the second EFA was conducted. The eigenvalues of the three factors were all greater than 1, the factor load was between 0.538 and 0.868, the item commonality was between 0.53 and 0.804, and all indicators were at a good level. According to the meaning of the items in each factor group, the factors were defined as factor 1 for skill, factor 2 for attitude, and factor 3 for knowledge (see Table 2 for details).

**TABLE 2:** Exploratory factor analysis of the scale of hotline counselors’ competence (*N* = 343).

Item	Coefficient of factor load			Commonality
	Factor 1	Factor 2	Factor 3	
q1			0.651	0.53
q2			0.666	0.593
q3			0.643	0.533
q4			0.786	0.695
q5			0.755	0.711
q6			0.749	0.724
q7			0.758	0.713
q8			0.619	0.61
q13			0.6	0.568
q14			0.538	0.609
q17	0.685			0.649
q18	0.696			0.637
q19	0.774			0.675
q20	0.75			0.653
q22	0.659			0.627
q23	0.68			0.587
q24	0.756			0.679
q25	0.72			0.682
q26	0.747			0.707
q30	0.693			0.638
q31	0.73			0.658
q32	0.689			0.592
q33	0.643			0.551
q34	0.635			0.609
q35	0.692			0.671
q36		0.72		0.573
q37		0.711		0.644
q41		0.713		0.62
q42		0.779		0.727
q44		0.809		0.702
q45		0.868		0.804
q46		0.756		0.659
q47		0.715		0.631
q48		0.731		0.645
q49		0.693		0.494
q50		0.705		0.58
q55		0.674		0.523
q57		0.656		0.601

### Item Analysis

The high-score group was the sample with a total score of ≥174. There were 94 samples in the high-score group, accounting for 27.4% of the total sample. The low-score group was the sample with a total score of ≤152. There were 101 samples in the low-score group, accounting for 29.4% of the total. An independent sample *t*-test between the two groups was conducted and found that there were very significant differences between the two groups in the scores of each of the 38 items (df = 193, *t* = 9.622 -22.731). The results indicate that each item had a high degree of differentiation and could distinguish the two groups significantly. Further analysis of the correlation between each item and the total score found that there was a very significant correlation between the scores of each of the 38 items and the total score, and all the correlation coefficients were greater than 0.5. This shows that the items in this scale were highly correlated with competence.

### Reliability and Validity Analysis of the Hotline Counselors’ Competence Scale

After eliminating invalid questionnaires based on the response time and completeness of the questionnaire, a total sample of *n* = 334 was obtained (M_age_: 43.7, S_age_: 9.48; female: 86.5%; education level: graduate: 38.3%, undergraduate: 52.4%, college diploma and below: 9.3%). The number of samples studied was more than eight times the number of items in the scale, which ensures the quality of the CFA.

#### Reliability Analysis of the Scale

The coefficient McDonald’s ω of each factor was 0.927 for Factor 1, 95% CI [0.914, 0.940], 0.958 for Factor 2, 95% CI [0.951, 0.965], and 0.954 for Factor 3, 95% CI [0.951, 0.965]. The Guttman’s half-reliability coefficient was 0.872. The scale can thus be considered to have good reliability.

#### Validity Analysis of the Scale

##### The structural validity of the scale

The CFA results in Table 3 show that, except for items q1–q3, q49, and q55, the standard load coefficients of other items were all larger than 0.7. In addition, the standard load coefficients for all items were significant at an α = 0.001 level. There was thus a good correspondence between the measurement items and the factors. The structural validity of the scale was good enough.

**TABLE 3:** Coefficient of factor load.

Factor	Item	(Coef.)	(Std. error)	*Z*	*P*	Std. estimate
Factor 1	q17	1	–	–	–	0.776
	q18	1.038	0.053	19.725	0	0.799
	q19	1.091	0.07	15.684	0	0.784
	q20	1.001	0.061	16.274	0	0.777
	q22	0.998	0.065	15.321	0	0.771
	q23	1.162	0.079	14.632	0	0.737
	q24	1.198	0.076	15.849	0	0.79
	q26	1.123	0.066	16.944	0	0.834
	q30	1.176	0.076	15.545	0	0.782
	q31	1.111	0.073	15.15	0	0.796
	q32	1.075	0.075	14.266	0	0.721
	q33	1.086	0.077	14.071	0	0.719
	q34	1.042	0.07	14.968	0	0.754
	q35	1.067	0.067	15.836	0	0.791
	q25	1.127	0.067	16.82	0	0.83
Factor 2	q36	1	–	–	–	0.745
	q37	1.187	0.065	18.24	0	0.828
	q41	1.076	0.072	14.916	0	0.792
	q42	1.182	0.074	16.031	0	0.84
	q44	1.076	0.069	15.659	0	0.826
	q45	1.129	0.068	16.632	0	0.869
	q46	1.217	0.078	15.516	0	0.818
	q47	1.112	0.073	15.312	0	0.81
	q48	1.093	0.071	15.298	0	0.806
	q49	0.707	0.062	11.331	0	0.616
	q50	0.957	0.069	13.823	0	0.738
	q55	0.961	0.077	12.451	0	0.672
	q57	1.047	0.075	13.916	0	0.74
Factor 3	q1	1	–	–	–	0.57
	q2	1.09	0.06	18.034	0	0.604
	q3	1.216	0.099	12.34	0	0.622
	q4	1.669	0.155	10.779	0	0.798
	q5	1.577	0.143	11.049	0	0.835
	q6	1.737	0.155	11.233	0	0.871
	q7	1.759	0.156	11.286	0	0.879
	q8	1.695	0.165	10.29	0	0.736
	q13	1.466	0.147	9.981	0	0.706
	q14	1.432	0.143	9.981	0	0.717

##### Differentiation of the scale items

Table 4 presents a comparison between the correlation coefficients of the three factors and the square root of each factor’s AVE. The square root of AVE of each factor was greater than the correlation coefficient of one factor and the other factors. This shows that there were large differences among the 38 items.

**TABLE 4:** Discriminant validity: Pearson correlation and AVE square root value.

	Factor 1	Factor 2	Factor 3
Factor 1	**0.747**		
Factor 2	0.727	**0.776**	
Factor 3	0.526	0.618	**0.783**

*The bold numbers are the values of the square root of AVE.*

##### The fit of the model

The ratio of chi-square to degrees of freedom, GFI, RMSEA, and other indicators of model fitting obtained by CFA are presented in Table 5. Obviously, except for the GFI being slightly lower than the criterion value of 0.9, all other indicators were in line with the criteria. This shows that the scale fit the model well.

**TABLE 5:** Model fitting indicators.

Common indicator	χ^2^	df	P	χ^2^/df	GFI	RMSEA	RMR	CFI	NFI	NNFI
Criteria	–	–	>0.05	<3	>0.9	<0.10	<0.05	>0.9	>0.9	>0.9
Value	1056.452	601	0	1.758	0.855	0.048	0.018	0.96	0.912	0.953

### Competencies of Psychological Hotline Counselors During the Epidemic

The competence of 334 participants was measured by using the competence model of psychological hotline counselors verified above. The average score for each dimension and the total were 4.13/5, 4.55/5, 3.87/5, and 4.21/5, respectively (see Table 6). The results show that the psychological hotline counselors scored high on this scale.

**TABLE 6:** Current competence of psychological hotline counselors.

	Max	Min	Average	Standard deviation
Skill	5	2.8	4.13	0.47
Attitude	5	3.15	4.55	0.42
Knowledge	4.5	2.7	3.87	0.42
Average score of competence	4.87	3.21	4.21	0.38

## Discussion

### Competence Characteristics of Psychological Hotline Counselors

This study developed a competence model of hotline counselors during major public health emergencies based on the three dimensions of knowledge, skills, and attitudes. It focused on competence in general psychological counseling, the psychological hotline service, and psychological assistance in public emergent events.

Similar to former research (Zhang, 2011; Lambie et al., 2018), this scale emphasized the fundamental elements of counseling theories and skills in the knowledge and skills dimensions; for example, “Obtained the basic knowledge of psychology” (F1, knowledge) and “Mastered the basic intervention skills” (F2, skills). Both of those research studies assessed the capacities of relationship building, empathy, and focusing compared with the scale developed by Liang et al. (2017). According to the general requirements of benevolence, responsibility, integrity, justice, and respect, Chinese Psychological Society (2018), items like “Treat callers responsibly” were included in the attitude dimension. Ethical practice was also assessed in the former scales (Zhang, 2011; Lambie et al., 2018).

The major specialty of this scale was that it reflected the specific requirements for hotline counseling that operated during the COVID-19 epidemic. As mentioned at the beginning of the paper, the hotlines normally provided time-limited (no more than 30 min) and single-session services. We also assessed related competencies with items such as “Ability to build a relationship with the callers effectively,” “Quickly focus on the major complaint of the callers and form the primary intervention plan,” and “Ability to identify and respond to emergencies and nuisance calls.”

The data analysis verified the original conception of the hotline counselor’s competence model, which has good reliability and validity. The model also shows that the hotline counselors have unique competency requirements, which cannot be replaced by general competency characteristics. The competence model requires specified items about the psychological hotline and psychological assistance.

### Professional Skills Are an Important Component of the Competence of Hotline Counselors

The EFA results show that professional skills have the highest contribution to the hotline counselor’s competence, which might be related to the specific context of psychological hotlines. People called for help due to distress and crisis after the initial stage of the outbreak and needed highly skilled counselors in psychological first aid and crisis intervention. Moreover, most psychological hotlines provided a single-session service with each session limited to approximately 30 min, which required the counselors to be able to quickly focus on the problems of callers and provide effective intervention. The results are also consistent with previous studies. Liang et al. (2017) compiled a skill evaluation form for hotline psychological intervention based on three dimensions: counseling process, counseling attitude, and communication skills. They used this form to evaluate the qualifications of the hotline counselors’ consulting skills, and emphasized the importance of consulting skills for the quality of hotline operation. From the perspective of competence training, operational experience is of fundamental importance for consulting psychology students to apply classroom learning in practice (Anderson and Ball, 1978; Weeks, 1982). The training process enables students to work on real clients under supervision and focuses on skill improvement (Brown, 1985). Skills had the greatest weight in constructing psychological hotline counselors’ competencies.

### Current Competence of Psychological Hotline Counselors

The psychological hotline counselors scored high on the self-assessment scale. This could probably result from the sample selection and the assessment goal of the scale. The questionnaire was distributed to major hotline organizations/platforms in China. Most of the participants in the study were hotline counselors who had been screened and recruited by the organizations/platforms, which set higher criteria for qualifications and experience. The participants were well trained and supervised. Also, this scale aimed at assessing minimum competency, and participants may have scored higher on the scale, especially when they were already experienced.

Zeng et al. (2014) investigated the competence of psychological counselors in colleges and universities, and also found that more than half of the participants scored over 4 points. However, Zhang’s survey on the competence of mental health professionals (2011) obtained the opposite results, which could be attributed to differences in the samples. In this study, the variation coefficients of scores on each dimension are close to that of the total scores, and the variation in scores on skills is the largest, which reflects that their competencies are on a par with each other. It can also be speculated that the difference in the total score mainly resulted from the difference in skills.

## Conclusion and Future Studies

The Psychological Hotline Counselor Competence Scale developed in this study has good reliability and validity. The scale is suitable for screening and assessing the competencies of professionals who provide psychological assistance via hotlines or other media after disasters or major public health emergencies. The scale could provide a convincing reference point for service organizations to assess and recruit competent professionals. It could also be utilized in the supervision of psychological assistance provided via hotlines. It could map out the strengths and weaknesses of supervisees. The supervisors could quickly identify the areas of incompetence and help the counselors to improve their competencies.

There are several limitations in this study. On the one hand, the questionnaire was developed during the COVID-19 epidemic, which was reflected in the specific requirements for psychological assistance provided via hotlines or Internet platforms. Thus, the utilization of this scale may be limited to the specific format of hotlines. In addition, this research was conducted in mainland China. The items selected also reflected the practical requirements for psychological assistance needed to address the issues that emerged during the COVID-19 epidemic. Thus, cultural differences should be taken into consideration when the scale is applied to other countries.

With the increasing use of this scale in the future, the continuous accumulation of samples would help to build a more stable and reliable norm for the competence of hotline counselors. Longitudinal follow-up research could also be conducted to analyze the changing pathways of counselors’ competencies and to explore related variables that may affect the competencies of psychological hotline counselors. This scale is also suitable for carrying out relevant intervention studies under major public health emergencies, for evaluating the effectiveness of supervision and training for psychological hotline counselors, and for customized training where there are weaknesses in areas of competence.

## Data Availability Statement

The raw data supporting the conclusions of this article will be made available by the authors, without undue reservation.

## Ethics Statement

Ethical review and approval was not required for the study on human participants in accordance with the local legislation and institutional requirements. Written informed consent for participation was not required for this study in accordance with the national legislation and the institutional requirements. Written informed consent was implied via completion of the survey.

## Author Contributions

All the authors were engaged in each stage of this research, including proposing the theoretical construct of competency model, developing the primary scale, distribute the link of questionnaire, running statistic analysis, and forming discussion. Online meeting was arranged once there was a progress.

## Conflict of Interest

The authors declare that the research was conducted in the absence of any commercial or financial relationships that could be construed as a potential conflict of interest.
